# Two Novel Mutations Associated With Ataxia-Telangiectasia Identified Using an Ion AmpliSeq Inherited Disease Panel

**DOI:** 10.3389/fneur.2017.00570

**Published:** 2017-10-30

**Authors:** Maria V. Kuznetsova, Dmitry Yu. Trofimov, Ekaterina S. Shubina, Taisiya O. Kochetkova, Natalia A. Karetnikova, Ilya Yu. Barkov, Vladimir A. Bakharev, Oleg A. Gusev, Gennady T. Sukhikh

**Affiliations:** ^1^Research Center for Obstetrics, Gynecology and Perinatology, Moscow, Russia; ^2^Institute of Fundamental Medicine and Biology, Kazan Federal University, Kazan, Russia; ^3^RIKEN Innovation Center, RIKEN, Yokohama, Japan; ^4^Preventive Medicine and Diagnosis Innovation Program, Center for Life Science Technologies, Yokohama, Japan

**Keywords:** ataxia-telangiectasia, Louis-Bar syndrome, *ATM* gene, prenatal diagnostic, IDP

## Abstract

Ataxia-telangiectasia (A-T), or Louis-Bar syndrome, is a rare neurodegenerative disorder associated with immunodeficiency. For families with at least one affected child, timely A-T genotyping during any subsequent pregnancy allows the parents to make an informed decision about whether to continue to term when the fetus is affected. Mutations in the *ATM* gene, which is 150 kb long, give rise to A-T; more than 600 pathogenic variants in *ATM* have been characterized since 1990 and new mutations continue to be discovered annually. Therefore, limiting genetic screening to previously known SNPs by PCR or hybridization with microarrays may not identify the specific pathogenic genotype in *ATM* for a given A-T family. However, recent developments in next-generation sequencing technology offer prompt high-throughput full-length sequencing of genomic fragments of interest. This allows the identification of the whole spectrum of mutations in a gene, including any novel ones. We report two A-T families with affected children and current pregnancies. Both families are consanguineous and originate from Caucasian regions of Russia and Azerbaijan. Before our study, no *ATM* mutations had been identified in the older children of these families. We used ion semiconductor sequencing and an Ion AmpliSeq™ Inherited Disease Panel to perform complete *ATM* gene sequencing in a single member of each family. Then we compared the experimentally determined genotype with the affected/normal phenotype distribution in the whole family to provide unambiguous evidence of pathogenic mutations responsible for A-T. A single novel SNP was allocated to each family. In the first case, we found a mononucleotide deletion, and in the second, a mononucleotide insertion. Both mutations lead to truncation of the ATM protein product. Identification of the pathogenic mutation in each family was performed in a timely fashion, allowing the fetuses to be tested and diagnosed. The parents chose to continue with both pregnancies as both fetuses had a healthy genotype and thus were not at risk of A-T.

## Introduction

Ataxia-telangiectasia (A-T), or Louis-Bar syndrome, which is associated with mutations in the *ATM* (ataxia-telangiectasia mutated) gene, is a rare hereditary disorder with multiple manifestations [OMIM 208900]. It is characterized by oculocutaneous telangiectasia, recurrent sinopulmonary infections, neurodegeneration, and progressive immunodeficiency (1–3). Characteristic laboratory and diagnostic findings include cerebellar atrophy, a high serum alpha-fetoprotein level, and chromosomal translocations involving immunoglobulin and T-cell receptor genes (4, 5). The manifestations of A-T appear in early childhood and include neurodegeneration manifesting as gait and truncal ataxia (6). In most cases, when both ataxia and telangiectasia are present, diagnosis of A-T is straightforward. However, the condition can be diagnosed earlier, after the onset of ataxia, but before telangiectasia becomes apparent, if confirmation by laboratory tests is obtained. Given that curative treatments for A-T are not available, early diagnosis is important for appropriate care and genetic counseling of families with an affected child (7, 8). Unfortunately, parents may not always notice the first manifestations of disease, and consequently they do not approach specialists for genetic counseling, delaying diagnosis of the child concerned. This, therefore, increases the risk of such families producing a second affected child.

The determination of pathological mutations in *ATM* is crucial for the early diagnosis of A-T. Since the full sequence of the *ATM* coding region (3,056 bp) was identified and published, many such mutations have been identified (9–15). The detection of *ATM* mutations is difficult because of the large size of the gene (150 kb divided into 66 exons), the fact that mutations are located throughout its length with no hotspots, and the difficulty in distinguishing mutations from polymorphisms. Consequently, molecular investigations of each new case of A-T must include full-length sequencing of *ATM* in one or more affected children. Verification of the *ATM* genotype in each family member is also necessary. Despite this, newly discovered pathogenic mutations are described each year, most of them resulting from investigations of affected families (16–18) or from *ATM* genotyping in local ethnic groups (19–21), including the Russian population (22).

Timely identification of mutations in *ATM* is particularly difficult for inhabitants of remote regions who usually present belatedly at specialized genetic counseling centers. Such families often ask for a consultation during an ongoing pregnancy, before a confirmed diagnosis in older affected children is available. To test for a pathogenic mutation in the fetus, a preliminary molecular screening of the affected and unaffected older children, as well as the parents, is required. Typical in this regard were the two families in this study who presented at our Research Center for Obstetrics, Gynecology and Perinatology in Moscow for genetic counseling. The first family had two affected children and the second family had only one. At the time of presentation, the mothers were pregnant. This imposed strict time limitations, since whether the fetal genotype is pathogenic or normal is the key information influencing decisions about continuation of the pregnancy.

### Family Case 1

A healthy 30-year-old woman presented at the Research Center for Obstetrics, Gynecology and Perinatology in November 2015 in the seventh week of pregnancy. The woman was in a consanguineous marriage with a 34-year-old man, her cousin. At presentation, the family had three children, two of them diagnosed with “Louis-Bar-like syndrome,” without molecular confirmation of mutation(s) causing this disorder. The oldest child, a 6-year girl, did not exhibit symptoms of any inherited syndrome.

#### Affected Children: Clinical Data

**Figure 1:**
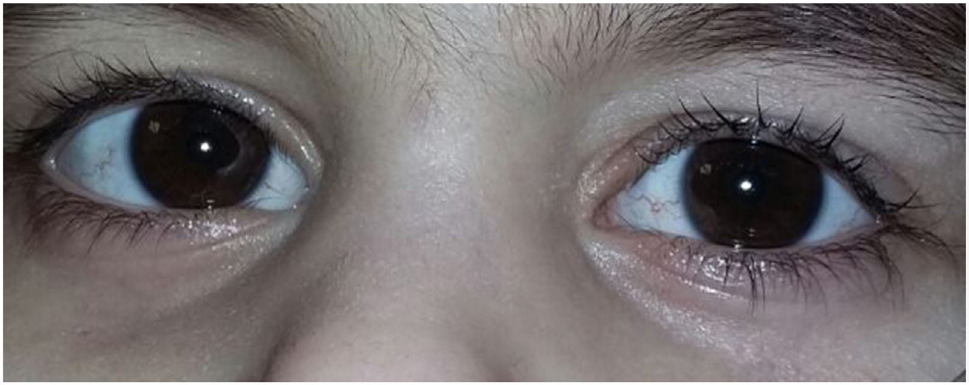
Manifestation of telangiectasia on the sclerae of both eyes in the affected child.

The first affected girl was born in 2010 after a full-term pregnancy without serious complications. During the postnatal period, she suffered from a number of respiratory conditions (bilateral pneumonia at the age of 1 year 7 months; chronic sinusitis, chronic obstructive bronchitis, pulmonary bronchiectasis). The girl started walking at the age of 3 years, her mother mentioned a staggering gait soon. A neurological examination at the age of 3 years revealed dysarthria and retardation in psychomotor development. Examination by a physician also revealed an oculocutaneous telangiectasia (Figure 1). Immunological investigations showed deficits in B-lymphocytes and both CD4 and CD8 T-cells, coupled with an elevated CD16 and CD56 NK-cell count. The levels of IgG and IgA in plasma were extremely low. An elevated α-fetoprotein level was detected.

This clinical picture, together with laboratory test data, led to a preliminary diagnosis of immunodeficiency of uncertain etiology, a chronic bronchopulmonary process, and retardation in psychomotor development. Various test data allowed mucoviscidosis, pulmonary malformations, and ciliary dyskinesia to be excluded. A diagnosis of Louis-Bar-like syndrome was postulated but not confirmed by laboratory testing.

The second affected child, a boy, was born in 2012. The pregnancy ran without serious complications and the child had no health problems during babyhood. However, once he started walking (3 years), the mother again noted a staggering gait. In 2014, the boy was investigated by several specialists, including immunologists and allergologists, in view of the known diagnosis of his sibling. The immunological survey revealed a similar situation to that of his affected sister, including an increased α-fetoprotein level and reduced levels of IgG and IgA. The neurological symptoms (ataxia) were considered to be a consequence of an ischemic lesion of the central neural system.

Taking into account the family anamnesis, the clinical findings, and the results of the laboratory tests, “Louis-Bar-like syndrome” was suggested as the most likely diagnosis for both siblings.

### Family Case 2

A 32-year-old woman originating from a North Caucasian region presented at our Center in April 2017 in the tenth week of pregnancy. The woman was in a consanguineous marriage with a 32-year-old man, her cousin. This couple had two children (born in 2012 and 2014), two stood pregnancies (2008 and 2009), one miscarriage (2016), and a current pregnancy (2017).

#### The Affected Child

The affected girl was born in 2012 in the 39th week of gestation. The pregnancy was accompanied by anemia in the mother and a high risk of miscarriage. In the post-natal period, the child suffered from a number of respiratory conditions (respiratory infections, pneumonia, meningoencephalitis, mononucleosis). In 2015, the girl was hospitalized with bilateral pneumonia. She was examined by a physician and an immunologist. A primary immunodeficiency with A-T was diagnosed and confirmed by laboratory tests. The immunological testing showed deficits in IgA, IgG, and CD4 T-cells. An elevated α-fetoprotein level was also detected. In March 2016, Louis-Bar syndrome was diagnosed.

Since the mother was pregnant, the family presented at the Research Center for Obstetrics, Gynecology and Perinatology for genetic counseling and prenatal diagnostics.

## Materials and Methods

### Tissue Samples and Genomic DNA Extraction

DNA was isolated from blood samples using the QIAamp DNA Mini Kit (Qiagen, Hilden, Germany) according to the manufacturer’s instructions. Samples of chorionic villus were treated with proteinase K for 3 h before DNA extraction. DNA concentrations were determined using a Qubit^®^ 2.0 Fluorometer (ThermoFisher Scientific, USA).

### Analysis of *ATM* Gene Using Ion AmpliSeq™ Inherited Disease Panel

To identify the pathogenic mutation in the *ATM* gene, we analyzed the affected child (a girl) from family 1 and the affected daughter from family 2 using the Ion AmpliSeq™ Inherited Disease Panel (ThermoFisher Scientific, USA). This kit provides highly multiplexed target selection of all exons and some introns of genes associated with >700 unique inherited diseases, according to the NCBI ClinVar database. In our case, we analyzed 60 coding exons of the *ATM* gene (two exons, 61 and 62, are not covered by the manufacturer, four exons are non-coding).

Sequencing libraries were prepared with the AmpliSeq Library Kit 2.0 (ThermoFisher) according to the user manual. Emulsion PCR was carried out with the Ion PGM Template OT2 400 Kit. Sequencing was performed on an Ion PGM machine using an Ion PGM Sequencing 400 Kit and an Ion 318 Chip v2.

Primary sequence data analysis (basecalling, trimming, filtering, and alignment to hg19 reference) was performed with Torrent server 4.4.3 software. Single-nucleotide polymorphisms (SNPs) were denoted as proposed by the Torrent Variant Caller 4.4 plugin. Annotation of alleles was performed using software developed in-house and dbSNP Build 150.

### PCR and Sanger Sequencing

For PCR amplification, 100 ng genomic DNA template was used. The following primers were used for PCR amplification of the regions of interest within the *ATM* gene.

Exon 48:

m48s1 5′-ggc agt tgg gta cag tca tgg taa-3′ (upper strand);
m48a3 5′-tga tga aaa gat gaa gca tat tca tgc-3′ (lower strand).

Exon 9:

atm9s1 5′-gtg ata cga gat cgt gct gtt cca-3′;
atm9a2 5′-ggt tga gat gaa agg att cca ctg aa-3′;
atm9s3 5′-acc aga tcc ttg gag att tct caa-3′;
atm9a4 5′-gga ttc cac tga aag ttt tct gaa-3′.

The samples were subjected to an initial heat denaturation at 95°C for 5 min, followed by 25 cycles of 95°C for 30 s, 64°C for 20 s, and 72°C for 40 s, and then 72°C for 30 min. The PCR products obtained were sequenced using the BigDye Terminator v.3.1 Kit (Applied Biosystems) bidirectionally on an ABI 3130 Automatic DNA Analyzer (Applied Biosystems, Foster City, CA, USA).

## Results

To locate the mutation directly responsible for the pathogenic phenotype, we sequenced 60 coding exons of the *ATM* gene (two exons, 61 and 62, are not covered by the manufacturer) in one of the affected children from each family using ion semiconductor sequencing, a next-generation sequencing (NGS) technology, and the Ion AmpliSeq™ Inherited Disease Panel. Several of the sequence variations detected in the two *ATM* gene sequences were benign (all of them previously described in dbSNP). However, the *ATM* gene in the affected child from family 1 harbored a formerly unknown single-nucleotide deletion in exon 48. This mutation was covered by 174 reads. It should cause a frameshift in the *ATM* mRNA and a truncation in the ATM protein. The sequencing data showed this mutation to be homozygous in the affected child, which could be readily explained by the close relation between the parents. The *ATM* gene in the affected child from family 2 harbored another formerly unknown mutation in exon 9, which was covered by 543 reads. This mutation was a single-nucleotide insertion [additional T in codon 408 (NP_000042.3)], causing a frameshift in the mRNA and probably a truncation in the ATM protein. Again, the sequencing data showed this mutation to be homozygous in the affected child.

### Sanger Sequencing

PCR amplification and Sanger sequencing were performed to verify the segregation of both mutations among the family members. The genotypes of all members of families 1 and 2 were investigated by complete sequencing of exons 9 and 48, respectively.

**Figure 2:**
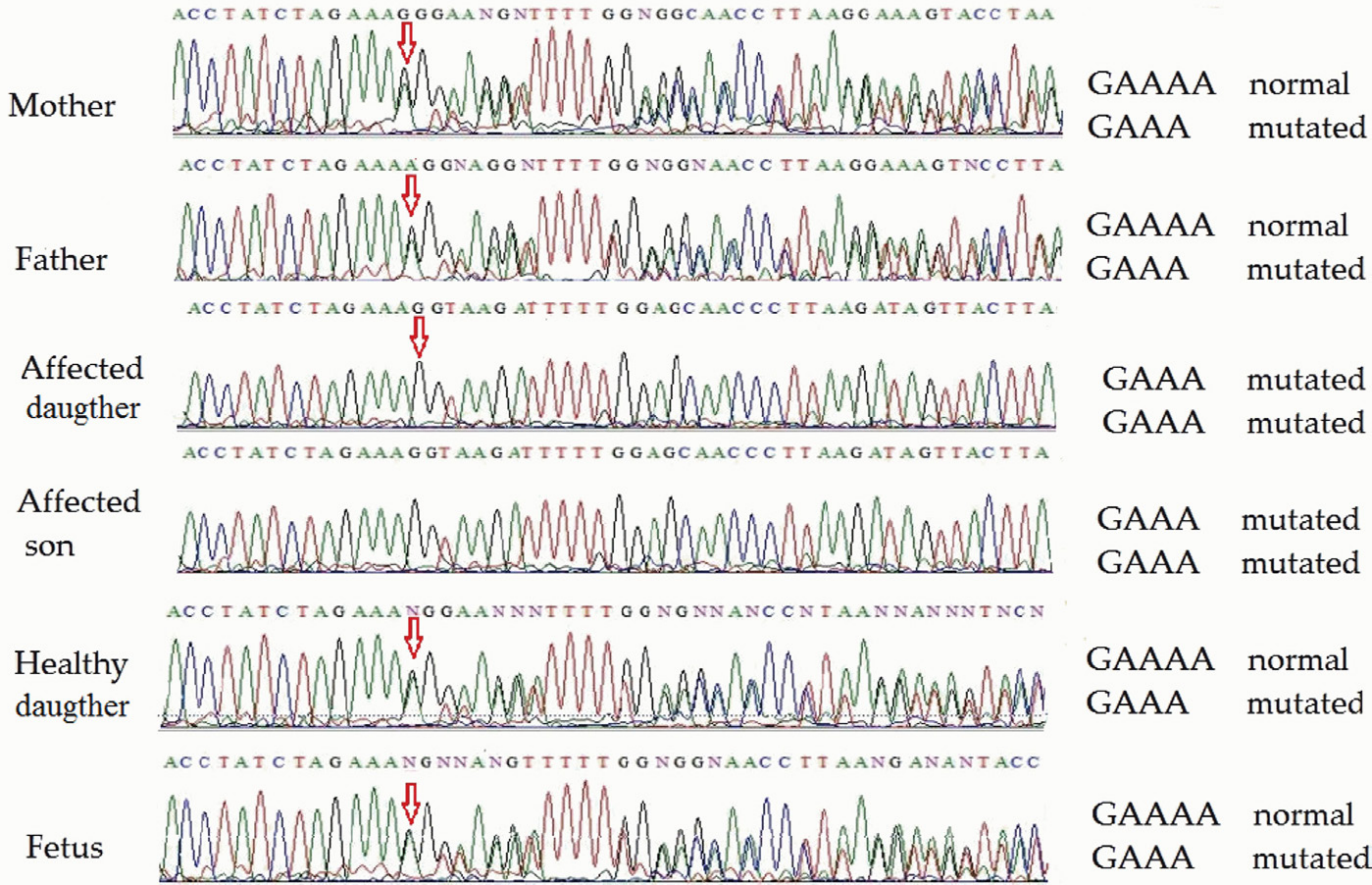
Partial *ATM* gene sequences determined by Sanger sequencing (upper strand read) of the members of family 1 (including fetus). The position of the mutation is shown by arrows. Heterozygosity is shown by double text to the right of the mutation (mother, father, child 3, and fetus), while homozygosity renders a readily readable single text (affected children 1 and 2).

Direct sequencing of *ATM* exon 48 (for family 1) on both strands revealed homozygosity of the novel single-nucleotide deletion in both affected children. Both parents, the healthy daughter and the fetus were heterozygous. Partial sequencing of exon 48 in all members of family 1 (including the fetus) is shown in Figure 2. This novel mutation was submitted to the ClinVar database,^1^ where it was registered under accession number rs876658512.

**Figure 3:**
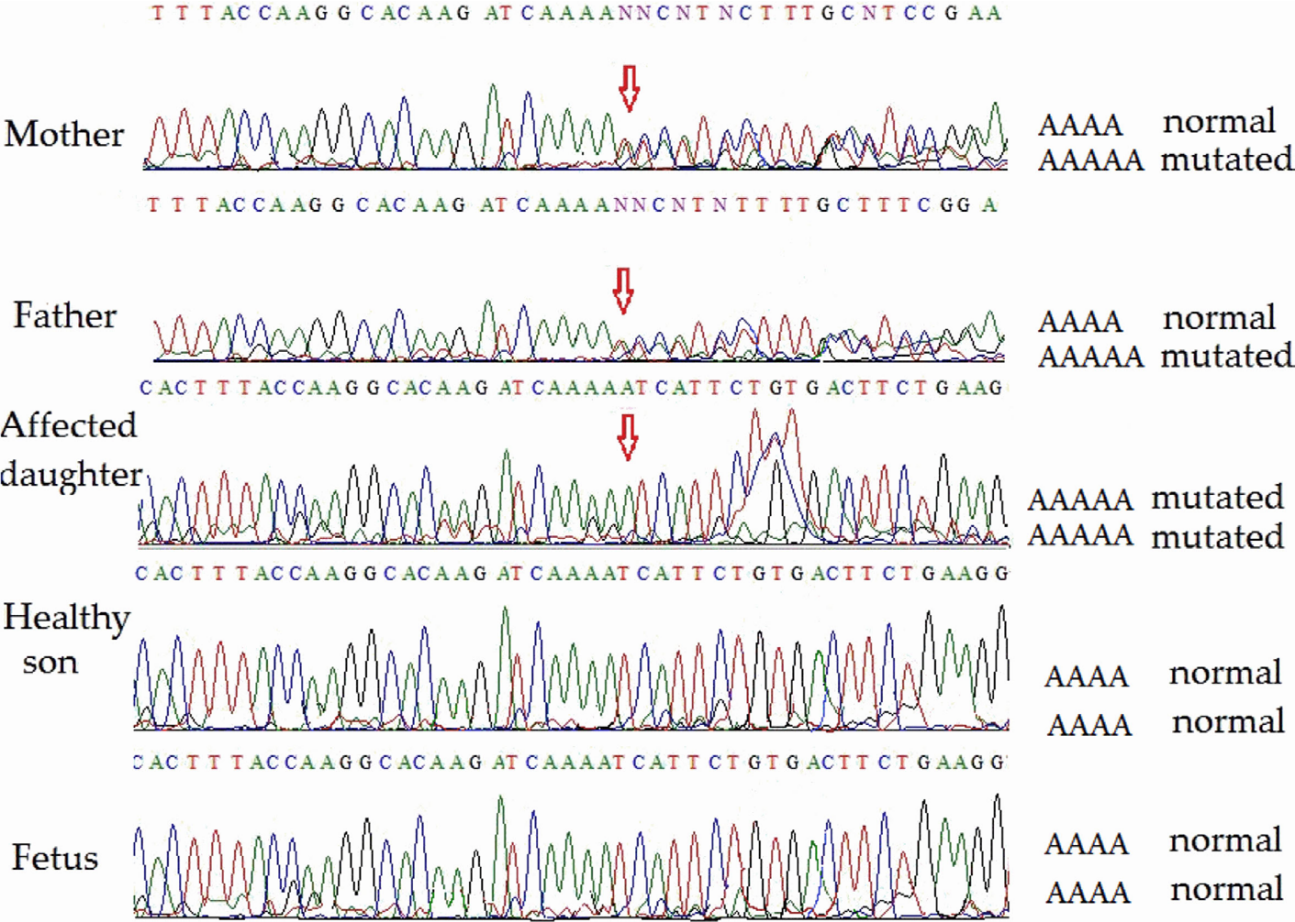
Partial *ATM* gene sequences determined by Sanger sequencing (lower strand read) of the members of family 2 (including fetus). The position of the mutation is shown by arrows. Heterozygosity is shown by double text to the right of the mutation (mother and father). The affected daughter is homozygous for the pathogenic mutation. The unaffected son and the fetus are homozygous for the normal allele.

Direct sequencing of ATM exon 9 (for family 2) on both strands revealed homozygosity of the novel single-nucleotide insertion in the affected child. Both parents were heterozygous, but the healthy son and the fetus were homozygous non-carriers of the pathological mutation. Partial sequencing results of exon 9 of all members (including the fetus) of family 2 are shown in Figure 3. This novel mutation has been submitted to the ClinVar database and given a temporary accession number (NM_000051.3); a permanent accession number will be available later.

The mutations reported here have been deposited in the Leiden Open Variation Database (LOVD).^2^ Registration IDs in dbSNP, ClinVar, and LOVD and some key characteristics of the mutations, are shown in Table 1.

**Table 1:** Annotated *ATM* gene variants affecting function in the investigated families.

Family	Exon	Type of variant	Variant (CDS position)	HGVS Name or NCBI rs ID	LOVD ID	Protein change (predicted)
1	48	Frameshift	c.7088delA	rs876658512	ID 00104957M ID 00104958F ID 00104959 AC1 ID 00104980 AC2 ID 00104981NA	p.(Lys2363Argfs3)
2	9	Frameshift	c.1221dup	NM_000051.3(ATM)	ID 00104982M ID 00104983F ID 00104984 AC	p.(Asp408 Terfs)

*M, mother; F, father; AC, affected child; NA, non-affected child*.

## Discussion

Over the last decade, NGS technology has made significant advances, especially with regard to prenatal diagnostics. Thus, a number of NGS-based investigations of different hereditary diseases have been reported (23–25). Molecular diagnosis of some inherited diseases (i.e., Gaucher disease, Duchenne/Becker muscular dystrophy, and some myotonic dystrophies) by conventional sequencing approaches is often precluded due to the large size of the genes of interest and the absence of evident hotspots suitable for rapid screening for known pathogenic mutations. However, NGS technology allows researchers and clinicians to obtain a full-length sequence of genomic fragments of interest (including long spans) for subsequent identification of novel mutations.

Due to the complexity of the experimental procedures and the degree of specialist, professional expertise required for such investigations, this type of analysis can be time consuming and is usually performed in reference molecular diagnostic centers. In the case of A-T, the situation is aggravated inasmuch as a diagnosis of the affected child might be delayed by such procedures.

It is well known that clinical diagnosis of A-T is difficult, especially at the early stages, when its manifestations can be masked by the normal variability among young children in achieving motor milestones. Together with the clinical heterogeneity of A-T symptoms, this often leads to a situation where the condition is recognized only at the age of 1–2 years (26, 27). According to Cabana et al. (7), the average age of A-T diagnosis (78 months) substantially exceeds the onset of gait abnormalities (15 months) and closely corresponds to the development of telangiectasia (72 months). The main manifestation of this syndrome is sensorimotor neuropathy, oculomotor apraxia, and progressive spinal muscular atrophy, mostly affecting the hands and feet. This results in affected children becoming wheelchair bound at the age of 10 years (28, 29).

Pathogenic mutations occur throughout the entire *ATM* gene and no hotspots or high-frequency haplotypes have been described (30). About 85% of typical, severe A-T cases are associated with homozygous mutations that lead to truncation of the ATM protein (31, 32). Despite the extensive array of previously described pathogenic mutations in *ATM*, each new family case requires full-length *ATM* sequencing, especially if the affected family belongs to a poorly investigated sub-population. Therefore, some authors have suggested that “mutation detection for A-T diagnosis is expensive and practically unaffordable”. However, the rapid progress of NGS technology has dramatically improved its clinical applicability. We chose ion semiconductor sequencing together with the Ion AmpliSeq™ Inherited Disease Panel as a comprehensive and efficient single assay strategy for screening for mutations in *ATM*.

**Figure 4:**
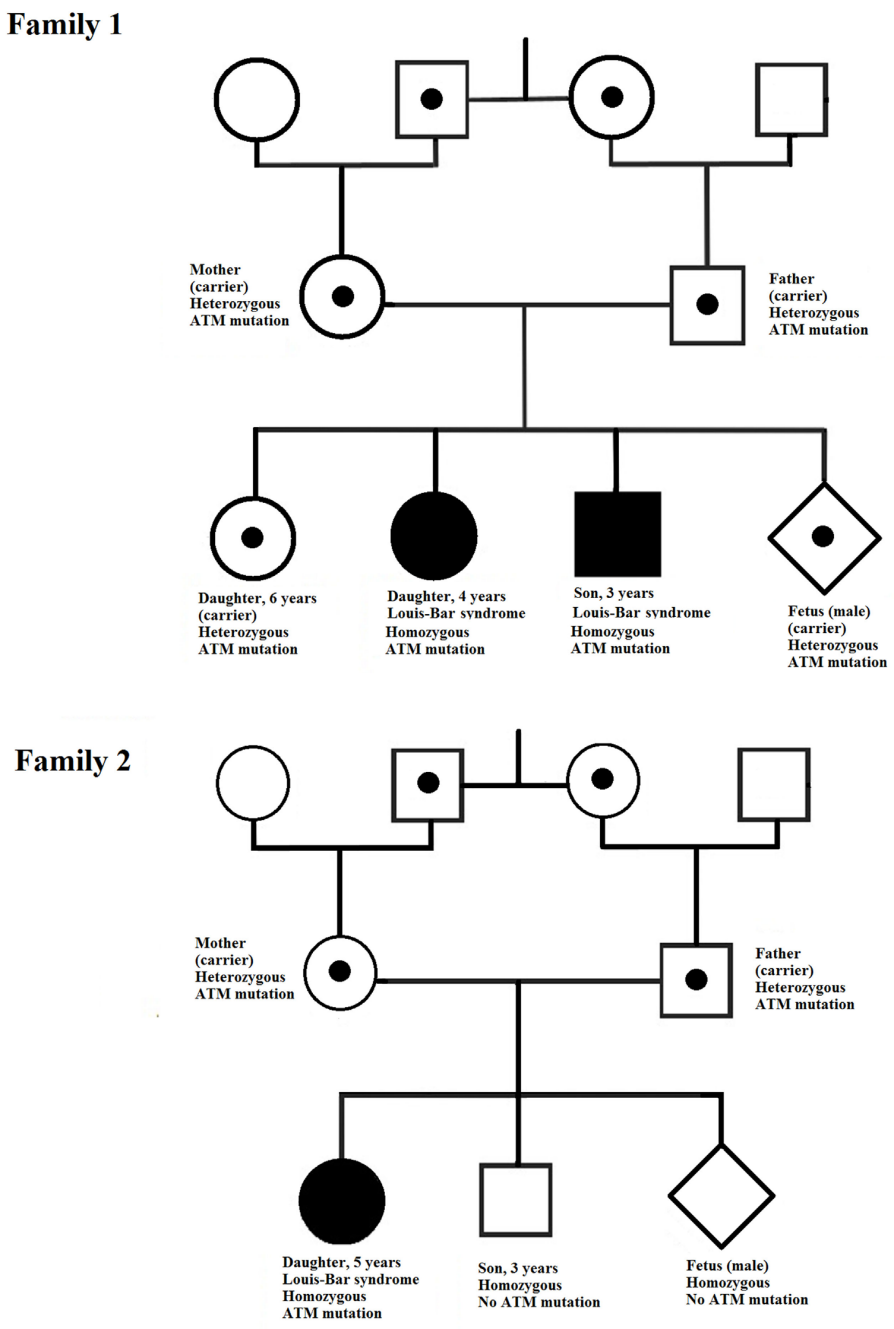
Pedigrees of the two families investigated. In each family, the *ATM* mutation is indicated by a point (in the case of heterozygosity) or a filled symbol (in the case of homozygosity).

In our study, the two affected children in family 1 exhibited early onset of ataxia, but telangiectasia was detected later. The age difference between these children is about 2 years and the onset of ataxia in the first child was followed closely by its onset in the second. The affected child in family 2 was diagnosed at 5 years of age. During these 5 years, the family had another baby, which was, fortunately, healthy. However, by the time of the next pregnancy, the first child had been diagnosed and rapid identification of the pathogenic mutation in *ATM* in this family was urgently required to allow prenatal diagnosis of the fetus. For both families, identification of the pathogenic mutations was performed in good time and the parents could choose to continue the pregnancy safely. The fetuses in both families had a healthy genotype and were not at risk of developing A-T (Figure 4).

Taking into account data of DNA analysis and clinical examination of the fetuses, both families obtained recommendations of genetic counselor of our Center about keeping the pregnancy.

## Conclusion

The advent of NGS technology provides new possibilities for medical diagnostics, including prenatal screening. This technology has recently developed to the stage where it is now applicable to routine clinical molecular diagnostic testing. In our case, it allowed in-time, accurate prenatal diagnosis of two fetuses with novel mutations (in exons 9 and 48) in the *ATM* gene. This allowed both families to make a choice in favor of continuing the pregnancy. A similar strategy should have broad applications, not only for diagnosis of A-T, but also for a wide range of monogenic diseases associated with long genes with numerous exons.

## Ethics Statement

Each individual participating in this study was informed and gave their written consent in accordance with the Declaration of Helsinki. We also obtained written consent from all participants for the publication of this manuscript. The study was approved by the Ethical Committee of Biomedical Investigations of the Research Center for Obstetrics, Gynecology and Perinatology, Moscow.

## Author Contributions

MK is the project coordinator. She has performed all amount of Sanger-sequencing and manual analysis of the sequences. DT is head of department. He was responsible for co-operation between groups of the physicians and molecular biologists within the team, provided on-time implementation of molecular tests in terms when pregnancy abortion is allowed. ES was responsible for computer analysis of NGS data. TK was responsible for NGS-data obtaining. NK carried out clinical examination of the patients. VB was responsible for genetic consulting of the patients (families). IB was responsible for administration of the project and management of laboratory sample collection. OG is a specialist in neurology. He provided a description of neurological anamnesis of the families. GS is a head of Research Center for Obstetrics, Gynecology and Perinatology. He was responsible for legal aspects of the project.

## Conflict of Interest Statement

The authors declare that the research was conducted in the absence of any commercial or financial relationships that could be construed as a potential conflict of interest.

## Footnotes

^1^http://www.ncbi.nlm.nih.gov/clinvar/variation/230341/.

^2^https://databases.lovd.nl/shared/genes/ATM.
